# Co-Occurrence of Multiple Risk Factors and Intimate Partner Violence in an Urban Emergency Department

**DOI:** 10.5811/westjem.2019.10.44190

**Published:** 2020-02-21

**Authors:** Raul Caetano, Carol B. Cunradi, Harrison J. Alter, Christina Mair

**Affiliations:** * Pacific Institute for Research and Evaluation, Prevention Research Center, Berkeley, California; † Highland Hospital - Alameda Health System, Department of Emergency Medicine, Oakland, California; ‡ University of Pittsburgh Graduate School of Public Health, Department of Behavioral and Community Health Sciences, Pittsburgh, Pennsylvania

## Abstract

**Introduction:**

Urban emergency departments (ED) provide care to populations with multiple health-related and overlapping risk factors, many of which are associated with intimate partner violence (IPV). We examine the 12-month rate of physical IPV and its association with multiple joint risk factors in an urban ED.

**Methods:**

Research assistants surveyed patients regarding IPV exposure, associated risk factors, and other sociodemographic features. The joint occurrence of seven risk factors was measured by a variable scored 0–7 with the following risk factors: depression; adverse childhood experiences; drug use; impulsivity; post-traumatic stress disorder; at-risk drinking; and partner’s score on the Alcohol Use Disorders Identification Test. The survey (N = 1037) achieved an 87.5% participation rate.

**Results:**

About 23% of the sample reported an IPV event in the prior 12 months. Logistic regression showed that IPV risk increased in a stepwise fashion with the number of present risk factors, as follows: one risk factor (adjusted odds ratio [AOR] [3.09]; 95% confidence interval [CI], 1.47–6.50; p<.01); two risk factors (AOR [6.26]; 95% CI, 3.04–12.87; p<.01); three risk factors (AOR = 9.44; 95% CI, 4.44–20.08; p<.001); four to seven risk factors (AOR [18.62]; 95% CI, 9.00–38.52; p<001). Ordered logistic regression showed that IPV severity increased in a similar way, as follows: one risk factor (AOR [3.17]; 95% CI, 1.39–7.20; p<.01); two risk factors (AOR [6.73]; 95% CI, 3.04–14.90; p<.001); three risk factors (AOR [10.36]; 95%CI, 4.52–23.76; p<.001); four to seven risk factors (AOR [20.61]; 95% CI, 9.11–46.64; p<001).

**Conclusion:**

Among patients in an urban ED, IPV likelihood and IPV severity increase with the number of reported risk factors. The best approach to identify IPV and avoid false negatives is, therefore, multi-risk assessment.

## INTRODUCTION

Intimate partner violence (IPV) includes acts of physical and sexual violence, stalking, and psychological aggression perpetrated against a romantic partner.^1^ This study, as have previous analyses of these data, 2,3 focuses on physical IPV. Community surveys have shown that about one in five couples in the United States (U.S.) have reported at least one episode of physical IPV in the prior 12 months.^4^–^6^ Data from the 2010–2012 National Intimate Partner and Sexual Violence Survey show 12-month rates for physical IPV of 3.9% among women and 4.7% among men.^7^ These rates are lower than those above likely due to differences in survey methods, especially telephone interviewing vs face-to-face, and interviews with one person only and not with both persons in the couple.

IPV screening in urban emergency departments (ED) shows rates ranging from 9–37% for a 12-month timeframe, and as high as 46% for lifetime exposure.^8^–^12^ A previous analysis of the data herein showed a rate of 23% for physical IPV, 4% for IPV perpetration only, 6% for victimization only, and 13% for mutual violence.^3^ Moderate and severe IPV were present in 12% and 11% of the sample, respectively, and about 48% of all IPV was severe.^2^ Identification of ED patients involved in IPV helps ED personnel to arrive at a better understanding of patients’ reasons for seeking care and to direct such patients to safe environments and support services.

The relatively high rate of IPV present among ED patients in urban settings has multiple causes. First, urban EDs are the entry point and sometimes the only setting for clinical care of health needs for a large part of the U.S. population that is socially disadvantaged, especially the 8.8% (28.3 million) without health insurance.^13^ Second, urban ED patients have high rates of substance use problems, unemployment, and depression,^14^–^16^ and are more often exposed to aspects of the social environment that are linked with IPV, such as neighborhood poverty.^17^,^18^ Third, ED patients report other IPV-related risk factors such as impulsivity, post-traumatic stress disorder (PTSD), partner hazardous drinking, adverse childhood experiences (ACE), and stressful life events.^2^,^10^,^19^–^24^ Finally, the ethnic composition of urban EDs includes a large proportion of disadvantaged ethnic minorities, some of whom are at higher risk for IPV.^9^,^10^

Examination of the association of risk factors and IPV in urban ED samples has focused on assessing the effect of each specific factor per se. However, ED patients may present with more than one risk factor, which suggests that it is also important to understand the potential cumulative effect on IPV risk when one, two, three, or more risks factors are reported by a patient. We examine the association between an index representing the cumulative effect of seven different risk factors and physical IPV. The risk factors composing the index are depression, PTSD, impulsivity, drug use, ACE, at-risk drinking, and partner hazardous drinking. Use of indices to create composite measures is a traditional practice in social and epidemiological research.^25^ There are two expectations guiding the analyses: a) IPV risk will increase as the number of risk factors increases; and b) IPV severity will also increase as the number of risk factors increases.

## METHODS

### Sample and Data Collection

Trained, bilingual (English and Spanish) research assistants (RA) recruited non-emergent patients in the ED of an urban Level I trauma center and county safety-net hospital. The initial sample size estimate called for the enrollment of 800 married, cohabiting, or dating adults aged 18–50. This was based on calculations that using linear regression analyses, power would be 80% to detect a small overall effect (*R*^2^ = .02) with 20 predictors, α = .05, and n = 800. Power would be 85% to detect small incremental changes of adding single variables to the regression equations (Δ*R*^2^ = .01) with 19 prior predictors, a prior *R*^2^ of .10, and α = .05.

**Population Health Research Capsule**

What do we already know about this issue?

Intimate partner violence (IPV) is highly prevalent in the U.S. population, with one in five couples reporting an incident in the prior 12 months.

What was the research question?

Does a combination of IPV risk factors increase IPV risk above the risk associated with one factor only?

What was the major finding of the study?

IPV rates increased substantially from 11% to 55% as risk factors present increased from one to four or more.

How does this improve population health?

Emergency department personnel should screen all patients for IPV, especially those presenting with multiple risk factors.

Participant eligibility criteria included the following: 18–50 years old; English or Spanish speaker; residence in the county where the study was conducted; and married, cohabiting, or in a romantic (dating) relationship for the prior 12 months. The upper age limit was set based on consistent research evidence showing that most IPV occurs in younger age groups.^26^ Patients who were intoxicated, experiencing acute psychosis or suicidal or homicidal ideation, were cognitively and/or psychologically impaired and unable to provide informed consent, in custody by law enforcement, or in need of immediate medical attention were excluded.

Two interviewers per shift staffed the ED during weekday peak volume hours (9 am– 9 pm) to recruit patients to the study. Data were collected from February through December 2017. Patients could opt to be interviewed in English or Spanish. We used a Spanish version of the questionnaire, which had been validated through translation into Spanish and re-translation into English, followed by verification. Once informed consent was obtained, patient survey data were collected by the RAs using computer-assisted personal interview (with computer tablets running the Qualtrics (Provo, UT, and Seattle, WA) platform. The project was approved by the institutional review board of the hospital where we conducted the study.

### Measurements

Reliability for the scales described below as measured by Cronbach’s alpha ranged from 0.69 for depression to 0.88 for perceived neighborhood disorder.^2^

#### Intimate Partner Violence

We measured prior-12 month physical IPV with the revised Conflict Tactics Scale,^27^ which has been used in prior ED-based IPV studies.^28^–^30^ Two levels of IPV severity, moderate and severe, were operationalized based on previously published reports.^31^ Moderate violence consisted of at least one of the following acts: threw something at partner that could hurt; pushed or shoved; grabbed; slapped; and twisted partner’s arm or hair. Severe violence consisted of kicked; punched or hit with something that could hurt; beat up; choked; burned or scalded on purpose; slammed against a wall; used a knife or gun.

#### Multi-risk Index

This is represented by the sum of seven IPV-related risks identified in previous analyses of this data set.^2^,^3^ Their assessment is described in detail below. These risks are depression, PTSD, impulsivity, drug use, ACE, at-risk drinking, and partner scoring positive on the Alcohol Use Disorders Identification Test- concise (AUDIT-C). Scores in the index vary from 0–7, but because few patients reported more than four risks as present, the variable was truncated at four or more risks.

#### Partner Problem Drinking

We used the three-item AUDIT-C to measure the respondent’s assessment of his/her spouse/partner’s drinking.^32^,^33^ Male partners with a score above 4, and female partners with a score above 3 in the test 0–12 scale were considered hazardous drinkers.

#### Drug Use

This measure covered drug use in the 12 months preceding the interview. Respondents were asked how many days they had used the following drugs: marijuana or hashish (without a doctor’s prescription); amphetamines; cocaine; heroin; and prescription pain relievers not prescribed for the user. Drug use was operationalized as any or no drug use.

#### At-risk Drinking

Respondents who drank alcohol in the prior four weeks were asked: “What was the greatest number of drinks you had on any day in the past 4 weeks?” A “drink” was defined as a 12-ounce can of beer, a five-ounce glass of wine, or a one-ounce shot of liquor. Respondents who did not use alcohol in the prior four weeks were asked the same question over the prior year. Women/men were considered at-risk drinkers if they had had four/five or more drinks on any one day in the prior four weeks (prior 12 months for prior year drinkers).

#### Adverse Childhood Experiences (ACE)

The modified ACE^34^ measures exposure to six adverse experiences during respondents’ “first 18 years of life”: 1) mentally ill person in the home; 2) parent/caregiver alcoholism; 3) sexual abuse; 4) physical abuse; 5) psychological abuse; and (6) violence directed against the respondent’s mother. These exposures are summed to create the ACE variable (range = 0–6). Scores in this variable were highly skewed, with 65% of the sample reporting none or one adverse experience. For inclusion in the multi-risk index in the analysis, this variable was operationalized as dichotomous representing none to one adverse experience vs two to six. Coding the variable as a dichotomy also allowed for a splitting of respondents that isolated the top tertile of the sample in the two or more group, which is the split applied to the impulsivity scale and the life stress scale described below. All of those with a score of two or more were included in the multi-risk index.

#### Impulsivity

This was measured with three items assessing respondents’ agreement with the following statements: I often act on the spur-of-the-moment without stopping to think; You might say I act impulsively; many of my actions seem to be hasty.^35^,^36^ Four response categories ranged from “not at all” to “quite a lot,” with scores ranging from one to four per item. For this analysis we divided scores into tertiles, and the scale was dichotomized with the two bottom tertiles coded as “none” and the top tertile coded as “one.”

#### Depression

This was measured with the Hospital Anxiety and Depression Scale,^37^ which has been successfully used in previous ED studies.^38^,^39^ Both anxiety and depression were measured with seven items each on a four-point Likert-type scale (eg, one = not at all; four = very often). The items request that respondents describe their “feelings currently.” Following Brennan et al.^40^ a cut-off point equal to or higher than eight identified positives. This cut off gives sensitivity of 0.82 and specificity of 0.74 for depression. The scale was dichotomized at the cut-off point for inclusion in the multi-risk variable.

#### Post-traumatic Stress Disorder (PTSD)

This measure is from the Primary Care PTSD Screen,^41^ and it too has been successfully used in ED studies (see^42^,^43^). It asks subjects about prior-month symptoms resulting from a “frightening, horrible or upsetting” experience. Answers were coded “yes” or “no,” and a score of three or more is considered positive.

#### Perceived Neighborhood Disorder (PND)

This was measured with Hill and Angel’s 10-item scale of neighborhood disorder.^44^ Items cover the extent to which assaults, muggings, drug dealing, gangs, unsafe streets, thefts, teenage pregnancy, abandoned houses, police not available, unsupervised children, and high unemployment, are neighborhood problems. Respondents could select one of the following three categories to answer each item: not a problem; somewhat of a problem; or a big problem.

#### Stressful Life Events

This was measured with 14 items from the Alcohol Use Disorder and Associated Disabilities Interview Schedule-IV.^45^,^46^ The items covered events such as the following: was laid off from a job; unemployed and looking for a job for more than a month; had trouble with boss or coworker; and had changed jobs, jobs responsibilities, or work hours. The items present were given a value of one and counted to create an index that varied from 0–14. Test-retest reliability is intraclass correlation = 0.94.^47^ For the present analysis scores were divide into tertiles, and the scale was dichotomized with the two bottom tertiles coded as “none” and the top tertile coded as “one.”

#### Other Sociodemographic Variables

*Gender:* A dichotomous variable coded as male and female (reference). *Age*: Coded as a categorical variable: 18–29, 30–39, and 40–50 (reference). *Level of education:* Respondents were categorized into four education categories: a) less than high school (reference); b) completed high school or GED; c) some college or technical or vocational school; d) completed four-year college or higher. *Importance of religion:* This variable had four categories – very important (reference); somewhat important; not very important; not important at all. *Marital status:* This is a three -category variable – a) married living with partner (reference); b) separated or divorced; c) never married. Widowers (n=33) were dropped from the analyses because 23 had no alcohol use disorder, which created estimation problems in the multivariable analysis. *Food insufficiency:* Respondents were asked their level of agreement with the statement, “In the past 12 months, the food we bought ran out and we didn’t have money to get more.” Response categories were never (reference), sometimes true, often true. *Ethnicity:* Based on self-identification. Respondents were asked: What racial or ethnic group(s) best describes you? Response categories were Asian; Black, African American; Latino, Hispanic (reference); White, Caucasian; Native American Indian/Alaskan Native; Native Hawaiian/other Pacific Islander; some other race (specify). Respondents who selected more than one category were identified as multiethnic.

### Statistical Analyses

We conducted all analyses with Stata 15.0 (StataCorp, College Station, TX).^48^ Associations in bivariate analyses (Tables 2 and 3) were tested with chi square. However, because the specific risk factors in each column of Table 3 are not mutually exclusive, the chi square tests differences in rates within each column, assessing first differences in the distribution of rates for any IPV vs none when a specific risk factor was or was not present. This was then repeated for differences in rates of no IPV, perpetration, victimization, and mutual violence, and for differences in rates of no IPV, moderate and severe IPV for each specific factor. Thus, we conducted a total of 18 chi-square tests (Table 3), which resulted in a Bonferroni corrected level of significance of .002 (.05/18) in that table.

We conducted multivariable logistic analysis (Table 4) with Stata’s “logistic” procedure. Independent variables were entered in the model in one step. Variables selection was based on previous analyses of the data set and previous results in the literature.^6^,^19^,^10^,^23^,^24^,^28^,^49^ We selected Hispanics as the reference group because they were the largest group in the sample (N = 520); this allowed for a contrast with Blacks, the second largest group (N = 299), and maintained consistency with a previous analysis focused on ethnicity and IPV.^2^ We conducted multivariable analysis of IPV severity (Table 4) with Stata’s “ologit” procedure, which implements an ordered logistic regression under a proportional odds assumption. Results indicated that the model tested fits the proportional odds assumption: chi^2^ = 9.05 with df =11 and p = 0.61. Therefore, only one set of adjusted odds ratios (AOR) are presented in Table 4. This is because the AORs represent both the odds of moderate plus severe IPV contrasted with no IPV, and the odds of severe IPV contrasted with no IPV plus moderate IPV.

## RESULTS

Missing data were negligible; none of the variables analyzed in this paper had more than 2.6% information missing. Thus, no imputation was conducted to address missing data, which were left as missing. We excluded from the study 34 ED patients who did not speak either English or Spanish.

### Sample Sociodemographic Indicators and Intimate Partner Violence Risk Factors

The sample is almost equally divided between men and women, with a mean age of 35.2 years (Table 1). About half of the sample is Hispanic, and about a third is Black. About a quarter of the sample did not report any of the seven IPV risk factors under analysis, and another quarter reported one risk factor.

### Intimate Partner Violence and Multi-Risk

About 48% of those who reported any IPV involvement experienced severe IPV (116/241), and of all IPV events reported, 16% were perpetration only, 26% were victimization only, and 57% were mutual violence. The proportion of all IPV reported by those with none, one, two, three, and four to seven risk factors is 4%, 13%, 23%, 19% and 40%, respectively. The proportion of all IPV reported by those with each specific factor under analysis is as follows: drug use, 60.2%; ACE, 49%; PTSD, 47.7%; impulsivity, 47.6%; partner AUDIT-C positive, 45.7%; at-risk drinking, 42%; and depression, 25.7%.

Results in Table 2 show that about a quarter of the sample reported at least one incident of IPV in the prior 12 months (rightmost column Table 2). The proportion of respondents reporting any type of IPV increases in a statistically significant way with the number of risk factors. Rates of IPV perpetration only, IPV victimization only, and mutual PV also increase in a statistically significant way as the number of reported risk factors increases. Rates of moderate and severe IPV also increase steadily with the number of risk factors.

### Intimate Partner Violence and Specific Risk Factors

Any IPV is present in 33% to 44% of respondents reporting the risk factors in Table 3. Rates of perpetration and victimization are lower than rates of mutual violence and do not vary much across respondents with any of the seven specific risk factors. Rates of moderate IPV are lower than rates of severe IPV for respondents reporting drug use, partner AUDIT-C positive, PTSD, and depression. Among respondents reporting impulsivity, at-risk drinking, and ACE, rates for moderate and severe IPV are similar.

### Correlates of Intimate Partner violence

The odds of reporting any IPV (first column of Table 4) increase with the number of risk factors. Blacks and multiethnic respondents are 1.8 and 2 times more likely, respectively, than Hispanics to report IPV. Finally, respondents who scored higher in the neighborhood social disorder scale are also more likely to report IPV. Mutivariable results for IPV severity are similar to results for any IPV.

## DISCUSSION

Both hypotheses put forward in the Introduction were confirmed: IPV risk and IPV severity increase as the number of risk factors reported by respondents increase. Rates for perpetration and victimization in Table 3 plateau when the number of risk factors reaches three. This may be because mutual IPV tends to be more severe,^2^ which means that it would be more strongly associated with three and four or more risk factors. Indeed, results in Table 2 show that the rate of mutual IPV among those with four or more risk factors is almost eight times higher than among those with one risk factor only.

But perhaps more importantly, respondents presenting with multiple risk factors may have IPV odds that can be six times higher than those with a single risk factor (Table 4). Further, assessment of one risk factor only may allow up to three quarters of IPV cases to go undetected. Given the high prevalence of IPV in ED populations and its numerous health-related consequences,^8^–^12^ the implication of these results is clear: assessment of multiple IPV risk factors is an important step to implement effective ED care in urban settings.

The two multivariable models in Table 4 confirm the results in previous tables with the added strength of controls for various potential confounders. IPV risk and severity increase in a stepwise fashion as the number of risk factors reported by patients goes from one to four or more. In addition, two other variables are important for the identification of subgroups with a higher prevalence of IPV: ethnicity and neighborhood disorder. Black and multiethnic respondents compared to Hispanics are about two times more likely to report IPV, which agrees with previous studies.^9^,^10^,^31^,^49^,^50^ The finding for the multiethnic group is challenging to understand because there have not been studies of IPV focusing on this population group in the U.S. The group comprised 6.9% of the U.S. population in 2015,^51^ while the Census Bureau’s estimate for people with “two or more races” in 2018 was smaller, 2.7%.^52^ Besides the proportion of persons, the share of mixed-race couples has increased since 1980 from 1.6% to 6.3% in 2013.^51^ This is a group that deserves more attention in epidemiological studies of IPV. Regarding perceived neighborhood social disorder, it can be associated with situations with lax behavioral norms and less informal social controls that minimize violence (eg, neighbors who call the police or intervene).^24^,^2^

## LIMITATIONS

The multi-risk variable only represents the additive effect of one or more risk factors on IPV. Non-additive effects were not tested but could be with the inclusion of an interaction term in multivariable models. However, the seven different risk factors in the analyses would result in 21 two-way interactions, and without a firm theoretical model to select which interactions to test, a decision was made to test additive effects first and in future analyses test interaction effects. The subjects enrolled were a convenience sample and may not be representative of the population. Results are from analyses of data from a single urban ED; thus, findings may not generalize to other EDs and other health settings. Also, the cross-sectional nature of the data does not support inferences about causation. In addition, recall bias may have affected subjects’ information about events that reached back over 12 months, and patient self-reporting of sensitive facts as IPV may lead to under-reporting.

## CONCLUSION

Results show that IPV risk factors co-occur in the same individual and that those who report the presence of two or more risk factors have increased odds of reporting IPV. These results, as those reported in a previous paper with a focus on ethnicity and IPV,^2^ help identify subgroups of urban ED patients that are more at risk for IPV and that should be the focus of specific IPV-related actions such as screening, brief intervention, or referral to treatment.

## Figures and Tables

**Table 1:** Sample characteristics: sociodemographic characteristics and intimate partner violence risk factors.

	% or M, SD
Sociodemographic characteristics	
Gender	
Male	46.6
Female	53.4
Marital status	
Married	40.2
Cohabiting	31.6
Single, separated, divorced	28.1
Education	
Less than high school	32.7
High school graduate/GED	35.5
Some college	22.4
College graduate+	9.4
Race/ethnicity	
Hispanic	49.2
Black	29.8
Multiracial	5.4
Other	9.2
White	6.4
Mean Age (range 18–50)	35.2 (8.5)
Food insufficiency	
Sometimes/often	49.6
Never	50.4
Number of risk factors	
None	23.0
One	25.3
Two	21.7
Three	12.4
Four or more	17.5
Specific IPV Risk Factors	
Adverse childhood experience (2+)	35.2
Drug Use (past 12 months)	33.0
At risk drinking (4+ women/5+ men)	28.0
Impulsivity (upper tertile score)	27.9
PTSD screen (positive)	25.1
Partner’s AUDIT-C (positive)	21.5
Depression (positive)	17.0

*GED*, general education degree; *M*, mean; *SD*, standard deviation; *IPV*, intimate partner violence; *PTSD*, post-traumatic stress disorder; *AUDIT-C*, Alcohol Use Disorders Identification Test-concise.

**Table 2:** Intimate partner violence (IPV) rates (proportions) by number of present risk factors in an urban emergency department sample.

	None (235)	One (259)	Two (225)	Three (129)	Four + (181)	Sample (1029)
% Any IPV***	3	11	26	37	55	23
Type of IPV***						
% Perpetration	1	2	4	8	7	4
% Victimization	1	4	10	9	9	6
% Mutual violence	1	5	12	20	38	13
IPV Severity***						
% Moderate IPV	3	8	16	19	21	12
% Severe IPV	1	3	9	18	34	11

Chi^2^

*** p<.001.

The statistical significance of distributions of perpetration, victimization, and mutual violence was tested with a chi square with df = 8. The statistical significance of distributions of moderate and severe IPV was tested with a chi square with df = 4.

**Table 3:** Intimate partner violence (IPV) rates (proportions) by specific risk factor in an urban emergency department sample.

	Drug use		Partner AUDIT-C positive		PTSD		Impulsivity		Depression		At-risk drinking		Adverse childhood experiences	

	No (695)	Yes (334)	No (809)	Yes (220)	No (775)	Yes (260)	No (745)	Yes (289)	No (860)	Yes (174)	No (745)	Yes (290)	No (671)	Yes (364)
% Any IPV	14	44*	19	44*	17	43*	17	41*	21	36*	19	36*	18	33*
% Perpetration	3	6*	4	5*	3	7*	2	7*	4	4*	3	6*	3	6*
% Victimization	5	9	5	12	5	11	5	8	6	8	6	7	6	7
% Mutual Violence	6	28	10	27	9	26	9	25	11	24	10	22	10	20
IPV Severity														
% Moderate IPV	9	19*	10	19*	9	20*	9	19*	12	15*	9	20*	10	16*
% Severe IPV	5	24	7	25	7	23	7	21	9	21	9	16	8	17

*AUDIT-C*, Alcohol Use Disorders Identification Test-consise; *PTSD*, post-traumatic stress disorder.

* All chi square no IPV x any IPV, no IPV x perpetration x victimization x mutual violence, and no IPV x moderate x severe p< .001.

**Table 4:** Multivariable logistic regression of any intimate partner violence (IPV) and ordered logistic regression of IPV severity on sociodemographic, drinking, and multi-risk variables.

	Any IPV		IPV Severity	

	AOR	95% CI	AOR	95% CI
Multi-risk (Reference: None)				
One	3.09**	1.47–6.50	3.17**	1.39–7.20
Two	6.26**	3.04–12.87	6.73***	3.04–14.90
Three	9.44***	4.44–20.08	10.36***	4.52–23.76
Four or more	18.62***	9.00–38.52	20.61***	9.11–46.64
Ethnicity (Reference: Hispanics)				
Black	1.85*	1.22–2.79	1.95**	1.29–2.93
White	1.29	.66–2.49	1.32	.69–2.53
Multiethnic	2.08*	1.05–4.10	2.00*	1.06–3.77
Other	1.77	.94–3.34	1.64	.86–3.14
Neighborhood Disorder	1.04**	1.02–1.08	1.04**	1.01–1.07

*AOR*, adjusted odds ratio; *CI*, confidence interval.

* p<05;

** p<.01;

*** p<.001.

Also controlling for gender, age, marital status, stressful life events, anxiety, importance of religion, education, and food insufficiency, none of which showed statistically significant associations. The weekly mean drinking volume was not statistically associated with IPV severity.
